# The Costs and Benefits of Risk Stratification for Colorectal Cancer Screening Based On Phenotypic and Genetic Risk: A Health Economic Analysis

**DOI:** 10.1158/1940-6207.CAPR-20-0620

**Published:** 2021-05-26

**Authors:** Chloe Thomas, Olena Mandrik, Catherine L. Saunders, Deborah Thompson, Sophie Whyte, Simon Griffin, Juliet A. Usher-Smith

**Affiliations:** 1 School of Health and Related Research, University of Sheffield, Sheffield, United Kingdom.; 2 The Primary Care Unit, Department of Public Health and Primary Care, School of Clinical Medicine, University of Cambridge, Cambridge, United Kingdom.; 3 Centre for Cancer Genetic Epidemiology, University of Cambridge, Cambridge, United Kingdom.

## Abstract

Population-based screening for colorectal cancer is an effective and cost-effective way of reducing colorectal cancer incidence and mortality. Many genetic and phenotypic risk factors for colorectal cancer have been identified, leading to development of colorectal cancer risk scores with varying discrimination. However, these are not currently used by population screening programs. We performed an economic analysis to assess the cost-effectiveness, clinical outcomes, and resource impact of using risk-stratification based on phenotypic and genetic risk, taking a UK National Health Service perspective. Biennial fecal immunochemical test (FIT), starting at an age determined through risk-assessment at age 40, was compared with FIT screening starting at a fixed age for all individuals. Compared with inviting everyone from age 60, using a risk score with area under the receiver operating characteristic curve of 0.721 to determine FIT screening start age, produces 418 QALYs, costs £247,000, and results in 218 fewer colorectal cancer cases and 156 fewer colorectal cancer deaths per 100,000 people, with similar FIT screening invites. There is 96% probability that risk-stratification is cost-effective, with net monetary benefit (based on £20,000 per QALY threshold) estimated at £8.1 million per 100,000 people. The maximum that could be spent on risk-assessment and still be cost-effective is £114 per person. Lower benefits are produced with lower discrimination risk scores, lower mean screening start age, or higher FIT thresholds. Risk-stratified screening benefits men more than women. Using risk to determine FIT screening start age could improve the clinical outcomes and cost effectiveness of colorectal cancer screening without using significant additional screening resources.

**Prevention Relevance:**

Colorectal cancer screening is essential for early detection and prevention of colorectal cancer, but implementation is often limited by resource constraints. This work shows that risk-stratification using genetic and phenotypic risk could improve the effectiveness and cost-effectiveness of screening programs, without using substantially more screening resources than are currently available.

## Introduction

Colorectal cancer (colorectal cancer) is the third most common diagnosed cancer in the world, accounting for 1.8 million new cases and 0.8 million deaths in 2018 (1). Screening is an effective way of reducing both mortality and incidence, by identifying colorectal cancer at earlier stages that are easier to treat and identifying and removing cancerous precursors (adenomas). Many high-income countries have chosen to screen their populations using the biennial fecal immunochemical test (FIT; ref. 2). The age at which FIT screening starts varies between countries with many starting at age 50 (e.g., Italy, France & Scotland; ref. 2). In England, FIT screening currently starts at age 60, despite studies indicating that reducing the start age to 50 would be highly cost-effective (3). While the intention is to reduce screening start age to 50 eventually, resource constraints mean that there is currently insufficient capacity (at the point of follow-up colonoscopy) to do this (4).

While age is the most important risk factor for colorectal cancer, many other genetic and phenotypic risk factors for colorectal cancer have been identified and quantified (5–8). In rare cases, individuals are at particularly high risk for colorectal cancer, for example, if they have monogenic conditions such as Lynch syndrome and familial adenomatous polyposis (9). Once identified, such individuals should receive specialist surveillance and are generally not included in population screening (10). For most individuals, colorectal cancer risk is due to a combination of a large number of risk and protective factors each contributing a very small amount. These include lifestyle factors such as alcohol consumption, physical activity, and diet (5), polygenic risk factors, of which more than 120 have been identified so far (7), and other characteristics such as family history and ethnicity, which are likely to be based on a combination of genetic and environmental risk (6, 8). Using various risk factors, a large number of different colorectal cancer risk-prediction models have been developed throughout the world (11, 12). Use of risk scores to determine who should be screened, and when, could result in better disease outcomes and more efficient targeting of scarce resources. Despite this, risk score–based screening has not been evaluated in practice and no current population-based screening programs use risk factors other than age to determine eligibility criteria.

While risk-based screening has not been evaluated in clinical trials, several recent modelling studies have examined the potential cost-effectiveness of risk-based strategies (13–15). However, these analyses have several limitations. First, they have been based on hypothetical risk scoring and have not evaluated or compared the many preexisting risk scores to determine whether any of these perform adequately. Second, none have evaluated risk scores that combine both genetic and modifiable phenotypic risk factors. Third, existing studies have generally not examined the benefits of risk stratification in the context of screening resource constraints that operate in practice and that prevent more cost-effective strategies using more screening resources, from being implemented.

Previously we reviewed published risk scores and externally validated them against UK Biobank data to determine which scores have the best discrimination within a UK population (16, 17). The aim of the analysis presented here was to use the best-performing risk scores including phenotypic and genetic risk as the basis for determining the age at which FIT screening should start, then to estimate the cost-effectiveness, clinical benefits, and resource impact of risk-stratification, compared with current screening strategies, from the English NHS perspective.

## Materials and Methods

A new individual patient-level microsimulation model was developed in R programming language: Microsimulation Model in Cancer of the Bowel (MiMiC-Bowel). This model simulates the life course of patients who each have a set of individual characteristics that determines their cancer risk and response to screening and surveillance. Patient baseline characteristics including phenotypic colorectal cancer risk factors are taken from the 6,787 individuals aged 30 or over in the Health Survey for England (HSE) 2014 (18). For this analysis, all individuals were set to age 30 at the beginning of the model and were then followed over time within the microsimulation. The model has a lifetime horizon and takes an NHS perspective.

### Model background

A brief summary of the model is presented here, with more detailed explanation available in an online technical methods document (http://eprints.whiterose.ac.uk/162743/). MiMiC-Bowel is comprised of several modules; a core natural history module, plus modules for symptomatic diagnosis, screening, and surveillance. In the natural history module, all patients are assumed to have normal colorectal epithelium at age-30 and patients then travel through nine different health states representing normal epithelium, low- and high-risk adenoma [definitions based on British Society of Gastroenterology (BSG) guidelines; ref. 10], colorectal cancer stages between A and D and death from colorectal cancer or other causes. The serrated adenoma pathway is represented by a transition directly from normal epithelium to colorectal cancer stage-A. Transitions between health states were derived through calibration to find parameter sets that enabled the model to replicate colorectal cancer incidence and prevalence of adenomas and undiagnosed colorectal cancer by age and sex in the absence of colorectal cancer screening (19–23). A full description of model calibration and tests for model validity are available online (http://eprints.whiterose.ac.uk/171343/). Transitions to colorectal cancer–related death were calculated from English colorectal cancer survival data from 2013–2017, which varies by age, sex, stage at diagnosis, and year from diagnosis (6). Other-cause–mortality was based on 2016–2018 English life tables by age and sex (24), from which known colorectal cancer mortality from 2018 death certificate data was subtracted (25).

Patients with colorectal cancer may be diagnosed symptomatically or through screening. Modeled screening procedures are based on the English Bowel Cancer Screening Programme (BCSP), with positive results at FIT leading to further investigation by colonoscopy or computed tomography colonography (CTC). Patients found to have adenomas undergo polypectomy and BSG guidelines are implemented in the model for surveillance following adenoma removal (10). Complications of endoscopy including perforation, major bleed, and mortality are included in the model (26, 27). Uptake of screening and follow-up procedures varies by age, sex, socioeconomic deprivation, and prior response to screening in line with published data (28, 29), but is assumed not to be altered directly by risk-stratification. The sensitivity and specificity of FIT screening, calculated by dividing screening detection rates by modeled disease prevalence, also varies by age, sex, FIT threshold, underlying disease status, and screening round (Supplementary Table S1; refs. 28, 30, 31). All modeled procedures are assumed to incur costs and resource use, with colorectal cancer treatment costs varying by age, stage at diagnosis, and year from diagnosis (32). All patients have an individual health-related quality-of–life which comes originally from HSE 2014 (18), but then is subject to decrements based on age, colorectal cancer diagnosis, and endoscopy complications. Potential psychologic impacts of screening are not included.

### Modeling stratified risk

Modeled phenotypic risk factors include body mass index (BMI), alcohol consumption, smoking, physical activity, and ethnicity. These were chosen as they are available from HSE 2014 for each modeled individual (18) and their relative risk for colorectal cancer has been reported (5, 6). Baseline values for lifestyle risk factors, and changes as individuals age, were incorporated in the model using a percentile method based on HSE 2014 data. Family history of colorectal cancer was not available from HSE 2014 but important for stratified colorectal cancer risk analysis (8). Both a “known” (age-dependent) and “actual” (age-independent) family history was assigned randomly to modeled individuals, based on proportions of the population with a first-degree relative with colorectal cancer at different ages in the UK Biobank population (33), with risk depending upon the “actual” family history.

Genetic data was incorporated by assuming that an individual's true genetic risk of colorectal cancer was represented by the 120 risk alleles associated with colorectal cancer, identified to date from Huyghe and colleagues (2019; ref. 7). Each modeled individual was randomly assigned each of these risk alleles, taking into account allele frequency obtained from UK Biobank data (33) and correlations between alleles on the same chromosome obtained from LDlink (34). Phenotypic and genetic risk factors were assumed to act independently on colorectal cancer risk, although in practice there are likely to be some correlations between them, particularly for family history and ethnicity. Phenotypic and genetic factors were combined for each person within the simulated population, to obtain a single individualized relative risk for colorectal cancer. These relative risks were applied to the transitions from normal epithelium to LR adenoma, representing the adenoma–carcinoma pathway, and from normal epithelium to colorectal cancer, representing the serrated pathway. Calibration was used to adjust relative risks to ensure the expected relative amount of colorectal cancer was occurring in people with and without risk factors.

To model the effects of using independent published risk scores to identify individuals for screening, two risk models identified from our previous work as having good performance were incorporated into MiMiC-Bowel; the Ma phenotypic only score (10) and the Jeon genetic only score (35), in addition to a score combining the two sets of risk factors (called MaJeon in this publication). Evaluation of these scores represents an independent assessment of the performance of risk-stratified screening. The discrimination of each risk score within MiMiC-Bowel, in a population cohort risk-assessed at age 40 and then simulated over a 10-year period, was assessed using the Area Under the Receiver Operating Curve (AUROC; Table 1; Supplementary Fig. S1). Discrimination is lower than previously found during validation against UK Biobank (16), as the impact of age predictors is absent due to use of a single-aged cohort in the model. In addition to using these independent risk scores, the performance of optimal risk scoring was also evaluated on the basis of genes alone (the Huyghe risk score), and the combined genetic and phenotypic factors included in development of the model (Huyghe plus BMI, physical activity, alcohol consumption, and smoking), with or without the additional incorporation of sex-specific risks (called “Total Risk” and “Total Risk + Sex” scores respectively in this manuscript). These were not independent from modeled colorectal cancer transitions, but enabled risk scores with higher discrimination to be assessed. Each risk score was adapted to enable calculation of absolute 10 year colorectal cancer risk based on Cancer Research UK colorectal cancer incidence for England (36). This enabled for example the Ma risk score, which is based on a Japanese population, to be calibrated to the English population.

**Table 1.** Characteristics of risk scores used in the analysis, based on assessing risk at age-40.

Risk score	Ma^a^	Jeon	MaJeon^a^	Huyghe	Total risk	Total risk plus Sex
Included risk factors	BMI	57 SNPs	BMI	120 SNPs	BMI	BMI
	Smoking		Smoking		Smoking	Smoking
	Alcohol		Alcohol		Alcohol	Alcohol
	PA		PA		PA	PA
			57 SNPs		120 SNPs	120 SNPs
						Sex
10-year AUROC: total	0.559	0.577	0.660	0.678	0.720	0.721
10-year AUROC: male	0.566	0.572	0.654	0.673	0.715	0.715
10-year AUROC: female	0.546	0.582	0.666	0.684	0.723	0.723
Proportion who get colorectal cancer in top decile of risk	0.145	0.157	0.242	0.250	0.314	0.316
Mean age at first FIT invite: with comparator age-60	60.06	60.11	60.50^b^	60.08	60.05	60.10
Mean 10 year risk: with comparator age-60	0.96%	0.98%	0.94%	0.90%	0.90%	0.86%
Mean age at first FIT invite: with comparator age-50	50.13	50.10	50.07	50.02	50.04	50.06
Mean 10 year risk: with comparator age-50	0.34%	0.34%	0.29%	0.32%	0.31%	0.30%

Abbreviations: BMI, body mass index; PA, physical activity; SNP, small nucleotide polymorphism; AUROC, area under the receiver operating characteristic curve; CRC, colorectal cancer; FIT, faecal immunochemical test.

^a^The original Ma score also includes age, but this was not included here as all individuals are being screened at age 40.

^b^The MaJeon age distribution is highly skewed compared to other scores meaning a risk level with a higher mean screening start age had to be selected to ensure a similar value for number of FIT invites per person.

Given that risk score administration costs are unknown and likely to vary considerably for different risk scores depending upon whether they require routinely gathered data, phenotypic data or genetic data, no costs were assigned to risk scoring itself. Instead, a justifiable cost analysis was carried out to determine the maximum justifiable cost of implementing risk-scoring in the population at age 40, while ensuring that risk scoring is still cost-effective. This is useful for policy makers thinking about using a risk stratified approach as it provides a guide as to how much could be spent on collecting risk information.

### Model analyses

The current FIT screening strategy in England [biennial FIT at a threshold of 120 μg/g (FIT120), age 60–74] was chosen as the basecase comparator. Three additional comparators were included; one with a start age of 50 instead of 60, a second using FIT20 rather than FIT120 as the threshold, and a third in which screening uptake was reduced by 25%, as the impact of risk stratification on uptake is unknown. The intention is for screening start age to fall to 50 eventually within the BCSP, and then for FIT threshold to be reduced when resources allow, and many other European countries currently start screening at age 50 and/or use a much lower FIT threshold than England.

Risk assessment was assumed to be carried out in all modeled individuals at age 40 using each of the risk scores described above. Age at first FIT invite was calculated for each individual as the age at which they would be expected to reach a particular risk threshold given their risk at age 40; the risk threshold was defined separately for each risk score to ensure that population mean screening start age allocated using that score, approximated comparator start age (Table 1; Supplementary Fig. S1). This was essential to ensure that total number of FIT screening episodes was kept at approximately the same level between comparator and intervention strategies, to ensure that any benefits of stratified screening could be attributed to the risk-stratification itself and not to reducing overall screening start age, or performing more screening overall. This is important because previous work has indicated that lowering screening start age below 60 in all individuals is cost-effective and reduces colorectal cancer incidence and mortality, but is currently not feasible in England due to resource constraints (3). Age at last FIT invite remained unchanged at 74 in all analyses.

Strategies were modeled using probabilistic sensitivity analysis (PSA), to enable parameter uncertainty to be incorporated. Discount rate was set at 3.5% for costs and QALYs in the basecase analysis as per UK National Institute of Health and Care Excellence (NICE) guidelines (37). Sensitivity analyses were carried out using alternative discount rates of 1.5% or 5%. Cost effectiveness was measured using incremental net monetary benefit [calculated as (incremental QALYs * willingness to pay threshold) – incremental costs], assuming a willingness to pay threshold of £20,000 or £30,000 per QALY (37). Outcomes were collected for the whole population and by sex. All model outcomes were weighted using HSE 2014 survey weights to represent the population of England.

### Data availability

Full description of model methods, parameters, and data sources are available online (http://eprints.whiterose.ac.uk/162743/). Model code is available on request.

### Ethical approval

This research has been conducted using the UK Biobank Resource under Application Number 28126. The UK Biobank study was conducted according to the guidelines laid down in the declaration of Helsinki and all procedures involving human subjects/patients were approved by the North West Multi- Centre Research Ethics Committee (reference number 06/MRE09/65). At recruitment all participants gave written informed consent to participate in UK Biobank and be followed up, using a signature capture device.

## Results

### Cost-effectiveness outcomes

**Figure 1.**
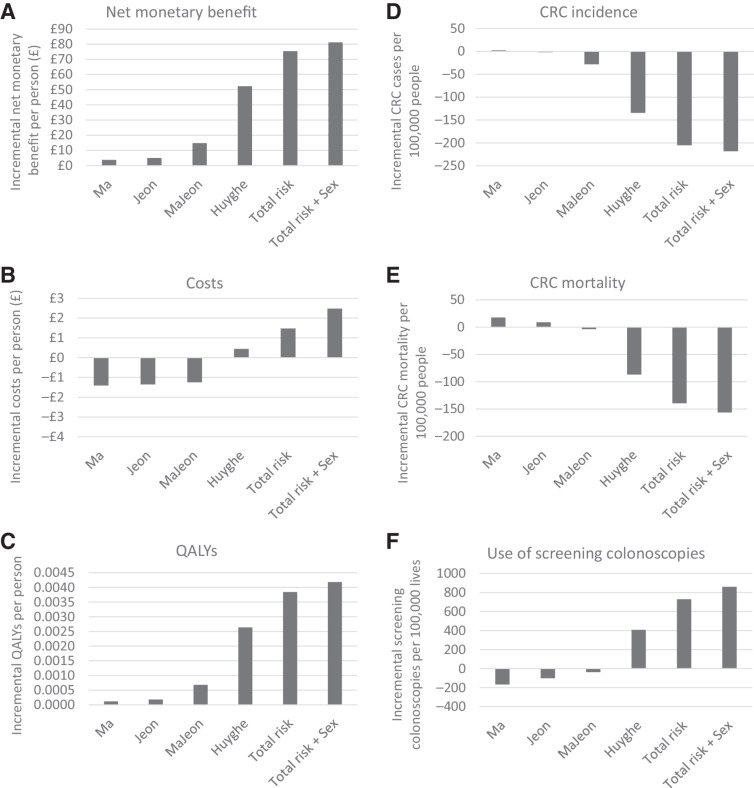
Incremental outcomes for risk stratification (based on a mean screening start age of 60), compared with screening start age of 60 for the entire population. Incremental net monetary benefit is based on a cost-effectiveness threshold of £20,000/QALY. **A:** Net Monetary Benefit, **B:** Costs, **C:** Quality-adjusted life years (QALYs), **D:** CRC incidence, **E:** CRC mortality, **F:** Use of screening colonoscopies.

Risk-stratification based on a mean FIT120 screening start age of 60 is expected to be cost-effective compared with starting FIT120 screening in all individuals at age 60, with incremental net monetary benefit per person ranging between £4 using the Ma score and £81 using the Total Risk + Sex score (Fig. 1; Table 2), assuming a willingness-to–pay threshold of £20,000 per QALY. Net monetary benefit is higher for the risk scores with higher discrimination. This difference in cost-effectiveness between risk scores is due to differences in QALY gain (ranging between 0.0001 QALYs per person using the Ma score and 0.0042 QALYs per person using the Total Risk + Sex score), as the higher discrimination risk scores also result in higher total costs, with some of the lower discrimination scores actually being cost-saving (ranging between -£1.41 per person using the Ma score and £2.47 per person using the Total Risk + Sex score). All scores result in reduced colorectal cancer treatment costs, with greater cost-savings coming from the high discrimination risk scores; however additional screening costs outweigh the colorectal cancer treatment savings for the higher discrimination risk scores (Table 2). In line with the cost-effectiveness results, the maximum cost at which risk scoring at age 40 could be priced, whilst still enabling the risk stratification strategy to be cost-effective at a threshold of £20,000 per QALY, varies between £5 per person for the Ma score and £114 per person for the Total Risk + Sex score.

**Table 2.** Full set of incremental outcomes for risk-stratification based on a mean FIT120 screening start age of 60, compared with screening everyone with FIT120 at age 60.

Risk score		Ma	Jeon	MaJeon	Huyghe	Total risk	Total risk + Sex
Total costs per person	M	−£1.41	−£1.35	−£1.24	£0.44	£1.47	£2.47
	*L*	−*£20*	−*£18*	−*£19*	−*£18*	−*£20*	−*£20*
	*U*	*£15*	*£15*	*£17*	*£19*	*£23*	*£22*
colorectal cancer treatment costs per person	M	−£2.11	−£1.73	−£3.37	−£6.38	−£8.55	−£8.51
	*L*	−*£21*	−*£20*	−*£21*	−*£26*	−*£31*	−*£32*
	*U*	*£15*	*£15*	*£16*	*£13*	*£14*	*£13*
Screen & surveillance costs per person	M	£0.70	£0.38	£2.12	£6.82	£10.02	£10.99
	*L*	−*£3*	−*£4*	−*£2*	*£2*	*£5*	*£5*
	*U*	*£5*	*£5*	*£7*	*£12*	*£16*	*£17*
QALYs per person	M	0.0001	0.0002	0.0007	0.0026	0.0038	0.0042
	*L*	−*0.0039*	−*0.0034*	−*0.0028*	−*0.0016*	−*0.0007*	−*0.0002*
	*U*	*0.0037*	*0.0037*	*0.0046*	*0.0072*	*0.0088*	*0.0094*
NMB per person (£20,000/QALY threshold)	M	£3.71	£4.99	£14.81	£52.27	£75.36	£81.15
	*L*	−*£70*	−*£64*	−*£57*	−*£29*	−*£12*	−*£4*
	*U*	*£76*	*£72*	*£90*	*£138*	*£177*	*£185*
NMB per person (£30,000/QALY threshold)	M	£4.86	£6.81	£21.59	£78.63	£113.77	£122.97
	*L*	−*£107*	−*£96*	−*£83*	−*£45*	−*£15*	−*£9*
	*U*	*£115*	*£109*	*£137*	*£211*	*£268*	*£275*
MJC per person (£20,000/QALY threshold)	M	£5.23	£7.04	£20.89	£73.74	£106.30	£114.48
	*L*	−*£99*	−*£90*	−*£80*	−*£41*	−*£17*	−*£5*
	*U*	*£107*	*£102*	*£126*	*£195*	*£250*	*£261*
MJC per person (£30,000/QALY threshold)	M	£6.86	£9.61	£30.45	£110.92	£160.49	£173.46
	*L*	−*£150*	−*£136*	−*£118*	−*£64*	−*£21*	−*£13*
	*U*	*£162*	*£154*	*£193*	*£298*	*£379*	*£389*
Prob. cost-effective (£20,000/QALY)		53%	55%	65%	89%	96%	96%
Prob. cost-effective (£30,000/QALY)		54%	55%	65%	89%	96%	96%
colorectal cancer incidence per 100,000 people	M	2	−2	−28	−134	−205	−218
	*L*	−*1117*	−*1035*	−*1127*	−*1275*	−*1384*	−*1391*
	*U*	*1109*	*1069*	*1062*	*1006*	*946*	*927*
colorectal cancer mortality per 100,000 people	M	18	9	−4	−87	−140	−156
	*L*	−*467*	−*459*	−*474*	−*568*	−*633*	−*647*
	*U*	*485*	*468*	*483*	*404*	*352*	*351*
FIT screening invites per person	M	0.016	−0.023	0.034	0.038	0.053	0.077
	*L*	−*0.008*	−*0.045*	*0.007*	−*0.003*	−*0.012*	*0.009*
	*U*	*0.041*	*0.000*	*0.062*	*0.076*	*0.110*	*0.136*
Screening colonoscopies per 100,000 people	M	−166	−101	−37	408	728	859
	*L*	−*847*	−*802*	−*710*	−*310*	−*49*	*14*
	*U*	*493*	*656*	*654*	*1143*	*1552*	*1655*

Abbreviations: CRC, colorectal cancer; L, lower 95% credible interval; M, mean; MJC, maximum justifiable cost (of risk scoring procedure); NMB, net monetary benefit; Prob., probability; QALY, quality adjusted life year; U, upper 95% credible interval.

Risk-stratification based on a mean FIT120 screening start age of 50, compared with starting FIT120 screening in all individuals at age-50, produces lower QALY gains (0.0001 to 0.0025 QALYs per person), higher total costs (−£0.34 to £3.82 per person), and consequently lower incremental net monetary benefit (£2 to £46 per person) and maximum justifiable cost of risk scoring (£4 to £65 per person) for each of the risk scores (Supplementary Fig. S2; Supplementary Table S2). In contrast, if FIT threshold is reduced to 20, but mean screening start age is kept at 60, the risk stratified approach produces slightly higher QALY gains (0.0001 to 0.0058 QALYs per person), all strategies produce cost savings (−£3.29 to −£1.96 per person), and incremental net monetary benefit and maximum justifiable costs for risk scoring are greater compared with FIT120 screening (Supplementary Fig. S3; Supplementary Table S3). As expected, reducing FIT uptake reduces the benefits of screening and the overall cost-effectiveness of each strategy (Supplementary Table S4). Reducing the discount rate results in lower incremental net monetary benefit for all risk-stratified strategies and vice versa for increased discount rates in most cases, although the Ma score is no longer cost-effective at a discount rate of 5% (Supplementary Tables S5 and S6).

**Figure 2.**
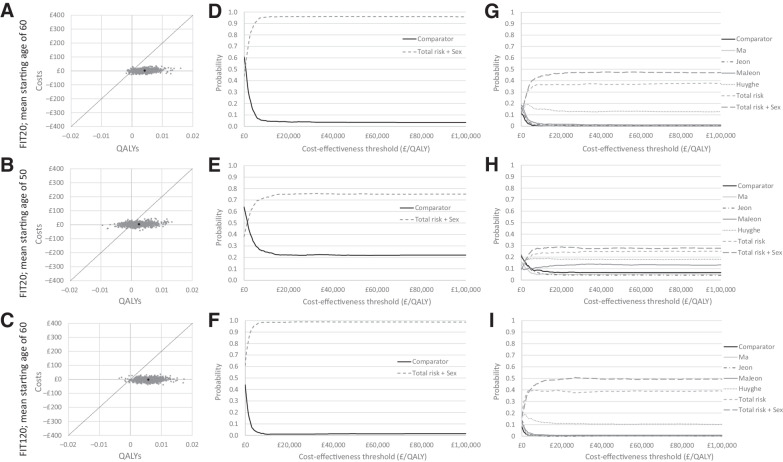
Cost-effectiveness planes (**A–C**) and cost-effectiveness acceptability curves (**D–I**) comparing probabilistic sensitivity analysis results for risk-stratification using the Total Risk + Sex score (based on a mean starting age of 60 or 50 and at a FIT threshold of 120 or 20), against inviting all at age 60 or age 50 respectively or against other risk scores.

Probabilistic sensitivity analysis results indicate that the best performing Total Risk + Sex score, when used for risk-stratification for FIT120 screening with a mean screening start age of 60, has a 96% probability of being more cost-effective than the comparator (all invited at age 60) and a 47% probability of being the most cost-effective risk score overall, assuming a willingness-to-pay threshold of £20,000 per QALY (Fig. 2). The Ma score in contrast has only a 53% probability of being cost-effective when compared with no risk stratification. Uncertainty is generally higher if the mean FIT screening start age is 50, with the Total Risk + Sex score having only 78% probability of being more cost-effective than the comparator (all invited at age 50), and 29% probability of being the most cost-effective risk score overall. Uncertainty is generally lower if a FIT threshold of 20 is used for screening, with the Total Risk + Sex score having 99% probability of being more cost-effective than the comparator.

### Disease & resource use outcomes

Risk-stratification using risk scores with low discrimination is expected to cause a slight increase in colorectal cancer incidence and mortality (e.g., an increase of 2 in 100,000 in colorectal cancer incidence and of 18 in 100,000 in colorectal cancer mortality using the Ma score with a mean FIT120 screening start age of 60; Fig. 1; Supplementary Table S2). By comparison, risk-stratification using risk scores with higher discrimination is expected to reduce colorectal cancer incidence and mortality considerably (e.g., a reduction of 218 in 100,000 in colorectal cancer incidence and of 136 in 100,000 in colorectal cancer mortality using the Total Risk + Sex score with a mean FIT screening start age of 60). Again, a clear gradient is apparent by risk score discrimination, smaller impacts are seen at when the mean FIT screening start age is 50 or if FIT uptake is reduced, and larger impacts are seen if the FIT threshold is 20 rather than 120.

The mean number of incremental FIT invites per person is very similar between all the strategies, as the intention was to minimize these differences when comparing strategies. FIT invites range from −0.023 for the Jeon risk score to 0.077 for the Total Risk + Sex score per person throughout their lifetime (Table 2). However, the number of screening colonoscopies varies quite considerably (between −166 per 100,000 people for the Ma score and 859 per 100,000 for the Total Risk + Sex score), reflecting the ability of each risk score to redirect screening resources towards higher risk people who are more likely to test positive with FIT screening. The combined impact of this differential screening resource use is to result in lower screening costs for scores with low discrimination and increased screening costs for scores with higher discrimination.

### Subgroup outcomes

**Figure 3.**
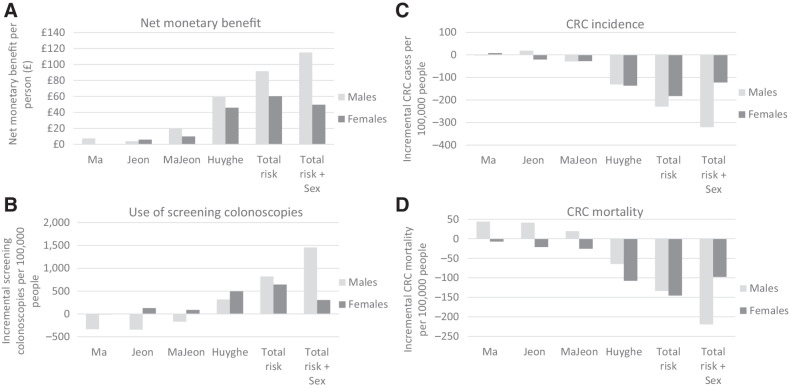
Incremental outcomes by sex for screening start age based on risk-stratification using the Total Risk + Sex score, compared with screening start age of 60 for the entire population. Incremental net monetary benefit is based on a cost-effectiveness threshold of £20,000/QALY. **A:** Net Monetary Benefit, **B:** Use of screening colonoscopies, **C:** CRC incidence, **D:** CRC mortality.

Benefits of risk-stratification are distributed unequally between men and women, particularly if sex is included as a criterion for risk stratification, but even where sex is not explicitly included (Fig. 3; Supplementary Table S7). Using the Total Risk + Sex score with a mean FIT screening start age of 60, the model predicts that men will gain more QALYs than women (0.0061 vs. 0.0024 per person), will lead to an increase in NHS costs, whilst women produce savings (£6.31 vs. −£1.11 per person), and compared with women, men will gain a bigger reduction in colorectal cancer incidence (−321 per 100,000 vs. −123 per 100,000) and mortality (−219 per 100,000 vs. −98 per 100,000). Due to the additional QALY gains, risk stratification is more cost-effective in men than women (incremental net monetary benefit per person is £115 in men, but only £50 in women) and the maximum cost at which risk scoring at age 40 could be priced, whilst still enabling the risk stratification strategy to be cost-effective at a threshold of £20,000 per QALY, is higher in men than women (£162 for men compared with £70 for women). Men are invited to FIT screening more frequently (an additional 0.93 invites), whilst women are invited less frequently (−0.72 additional invites), and whilst the number of screening colonoscopies increases in both sexes, this is much greater in men than women (1455 additional colonoscopies per 100,000 men vs. 303 per 100,000 women). This indicates that part of the benefit of risk-stratification in men is due to a redistribution of resources away from the women at lower risk, to the men at higher risk, although both sexes are expected to benefit overall from improved health and cost-effectiveness outcomes.

## Discussion

Our findings suggest that stratified screening in which individuals are invited to screening based on personalized risk, assessed through genetic and/or phenotypic risk scores rather than age alone, is likely to save costs and reduce colorectal cancer incidence and mortality without significantly increasing resource use. Given the resource constraints currently preventing cost-effective initiation of screening at younger ages in many countries including England, this could represent a reasonably resource-neutral means of improving efficiency, providing that costs of risk scoring can be minimized. However, we have shown that these benefits of risk-stratification diminish if resources are available to screen the entire population at a younger age. This result is supported by evidence from other modelling studies that have compared strategies in which screening starts from age-50 or younger, and which have concluded that risk stratification based on genetic risk is not likely to be more cost-effective than uniform screening at current levels of risk score discrimination (13, 14). This means that whilst risk-stratification may be an attractive option currently in England where population screening starts at age 60, it may not be attractive in other countries that start screening at lower ages, or in England in the future if more resources become available for screening.

The results indicate that benefits increase as the discrimination of the risk scores used increases. Scores with low discrimination such as the Ma score that has an AUROC of 0.56 appear to be cost-effective primarily through their impacts in increasing cost savings due to screening lower risk people later/less frequently, producing few or even negative health benefits. However, higher ranking risk scores with AUROCs of above 0.65 are likely to promote QALY gains in addition to cost-savings, and result in colorectal cancer incidence and mortality reductions. In external validation within a UK cohort, the risk score by Huyghe with 120 SNPs had an AUROC of 0.62 (95% CI, 0.61–0.64) in both women and men (16), which is close to this level, although the performance of this score could not be evaluated independently within our model. It is important to note that discrimination of risk scores may differ considerably between cohorts from different countries, and scores that perform well in a UK population may not rank as highly in non-European populations, indicating that there may be a need for different risk scores with high discrimination for different populations of interest. With increasing access to large datasets and more sophisticated statistical techniques, it is likely that better performing models will be developed in the future that combine known genetic and phenotypic risk for specific populations.

The costs of calculating risk in the population at age 40 were not incorporated within the modelling as these currently cannot be accurately quantified. However, the maximum justifiable cost analysis suggests that this could be as high as £114 per person for risk scores with high discrimination and risk-stratification, whilst still being cost-effective. Actual costs of risk-scoring will vary depending upon whether included risk factors are already routinely collected in primary care, or whether additional data collection or sampling (e.g., for genetic testing) is required and if so, whether these additional costs can be offset by combining them with data collection for other screening programs. The relative cost-effectiveness of different risk scores is therefore highly likely to differ, dependent upon their administration costs as well as the gradient of benefits they produce. In addition to the cost, there are also logistical challenges associated both with linking routinely collected primary care data with screening program data and collecting new information on the population. In England the NHS Health Check program provides a potential vehicle for collecting new information. At age-40 all those without a prior history of cardiovascular disease are invited to their first 5-yearly health check (38), which involves taking blood samples and collecting information about phenotypic risk factors including BMI and smoking. It seems feasible that this NHS Health Check could be extended for fairly minimal cost, to enable collection of additional risk factor information and assessment of risk for colorectal cancer and potentially other conditions. However, uptake of NHS Health Checks is currently only 41% (39), meaning that the risk for many people would not be assessed. An alternative route would also be required for those not eligible for an NHS Health Check. In this analysis we have assumed that the risk of all individuals is known; however, incomplete uptake would significantly reduce the overall population benefits of risk-stratification. This analysis has also assumed that individuals would not change either their screening uptake or their colorectal cancer risk behavior in response to risk-assessment, although there is some evidence that willingness to uptake screening could be increased in people who know they are high risk, and unchanged in people who know they are low risk, which if true could improve cost-effectiveness of risk stratification (40). Using risk-scoring as an opportunity to modify lifestyle risk factors could increase its cost-effectiveness further if costs of behavior-change–interventions could be minimised. In England, some behavior change programs that would reduce colorectal cancer risk (e.g., smoking cessation and weight management) are already offered to some people as part of the NHS Health Check (38), and uptake or effectiveness of these might be improved if patients were also aware of the potential impact on their colorectal cancer risk, although evidence from a recent study suggests that there is little impact on health-related behavior in response to risk scoring (41). Uptake of risk-scoring and behavior change in response to risk-scoring should be investigated as part of any future pilot study for risk score-based screening.

Our results suggest that inviting individuals to screening based on estimated risk rather than age alone, is likely to benefit men more than women due to the higher underlying rate of disease in men (42), and also that they will start screening earlier on average than women when risk scores including sex, or phenotypic risk factors correlated with sex, are used. This results in a shift of screening resources towards men, and greater improvement in their colorectal cancer incidence and mortality outcomes. Whilst uptake of screening is lower in men (43–46), the modelling indicates that overall, the benefits of inviting them at a younger age outweighs the disadvantages of not inviting women until older age, thereby both improving efficiency and mitigating some of the inequities inherent to colorectal cancer screening. This analysis also only focused on one potential application of risk-stratification – to determine age at first FIT screen, but risk stratification could be applied elsewhere in the screening pathway, for example, using a combination of individual colorectal cancer risk and FIT score to determine who gets follow-up colonoscopy, or using a combination of individual colorectal cancer risk and result of previous screen to determine when the next screening invite should occur.

There are several limitations of the modelling that could impact on results. Data limitations have meant that some known risk factors are missing from the model (in particular red meat and fibre, neither of which are in HSE 2014; ref. 18), and correlations between the set of correlated genetic risk factors and the HSE correlated phenotypic risk factors are absent. Assumptions were also made that all risk factors will impact on the first natural history transitions from normal epithelium only, and through both the adenoma carcinoma and serrated natural history pathways to a similar extent, thereby making disease attributed to all risk factors equally amenable to screening. Whilst there is no data to inform this, it is unlikely to be the case given the posited differences between pathways and adenoma/colorectal cancer prevalence in terms of age and sex distribution and location in the bowel. Data limitations have also resulted in high uncertainty around modelling results. Better quality routine data collection (e.g., using FIT screening data from the BCSP now it has commenced in England, rather than FIT pilot data), and more knowledge around colorectal cancer natural history should enable more accurate estimates of the benefits of risk-stratification to be produced in the future.

## Supplementary Material

Supplementary Data
