# Supplementary material for: The Costs and Benefits of Risk Stratification for Colorectal Cancer Screening Based On Phenotypic and Genetic Risk: A Health Economic Analysis
Source: Cancer Prev Res (Phila). 2021 May 26;14(8):811–22. doi: 10.1158/1940-6207.CAPR-20-0620 (PMC7611464; doi:10.1158/1940-6207.CAPR-20-0620)
Supplement: Supplementary Data [file 256247_2_supp_7042872_qr9kpz.docx]

Supplementary Figure 1: Receiver Operating Curves and FIT starting age distribution (based on a mean starting age of 60) for each risk score, based on assessing 10-year risk at age 40.


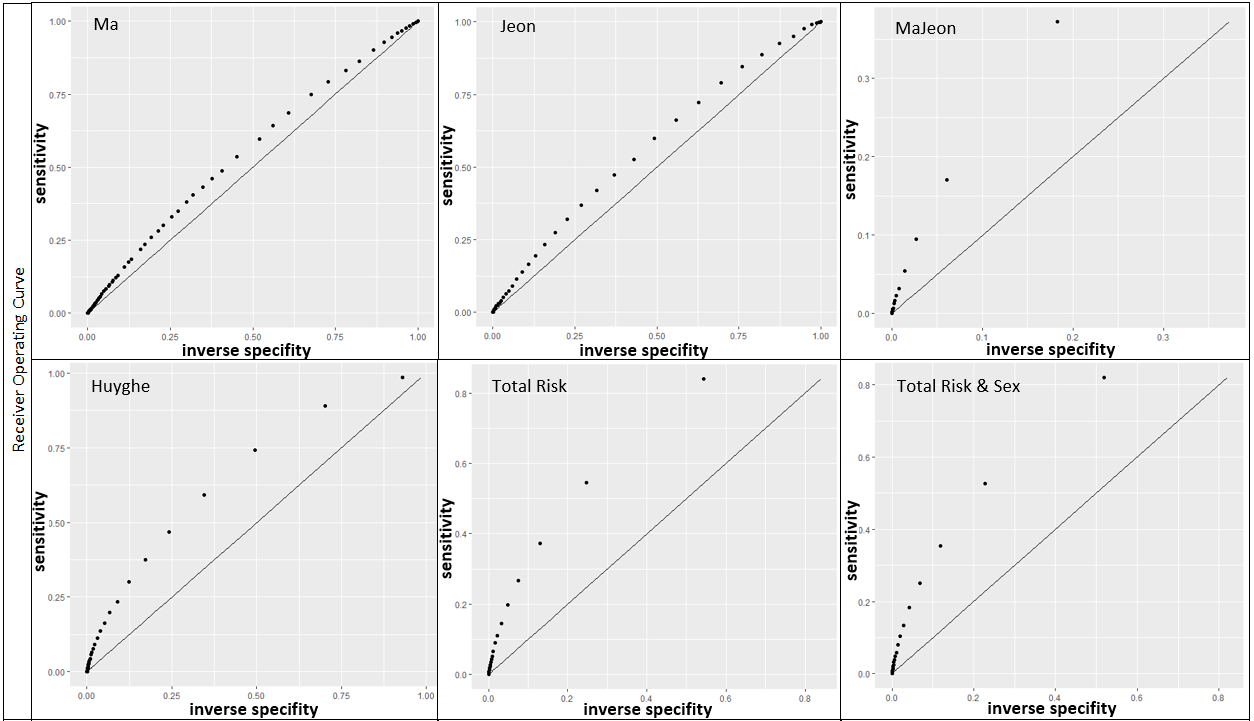

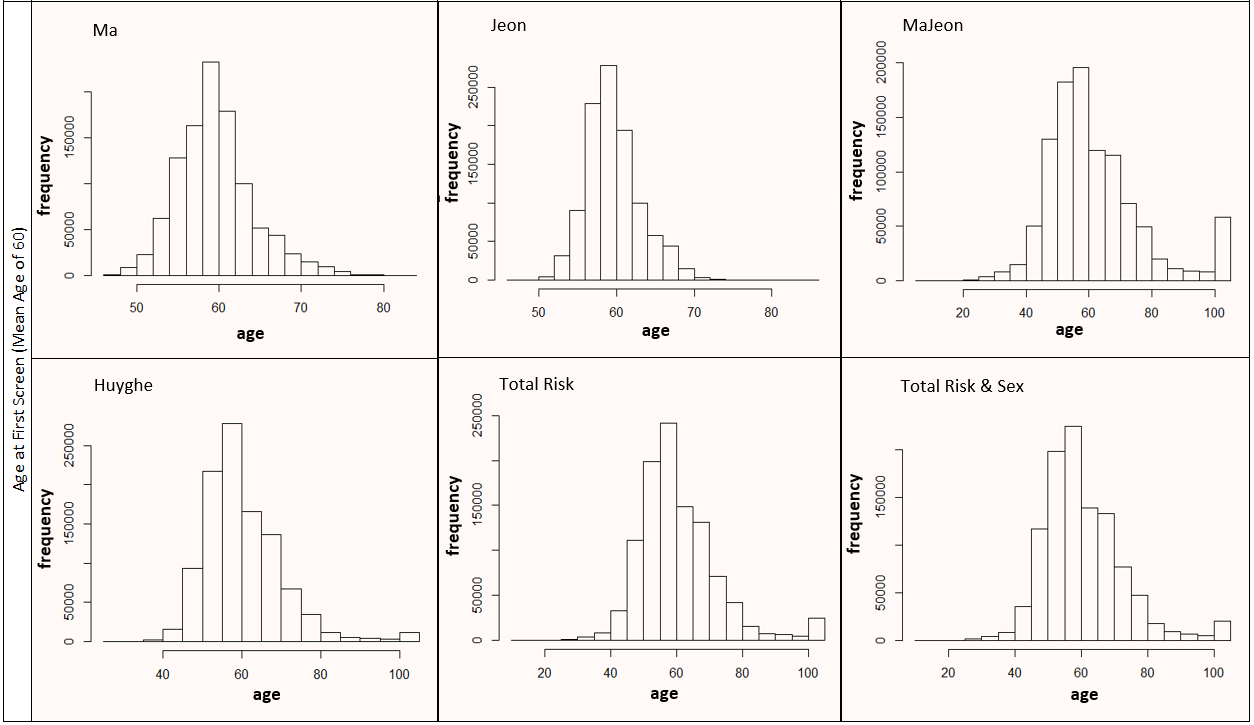


Supplementary Figure 2: Incremental outcomes for risk-stratification (based on a mean screening start age of 50), compared to screening start age of 50 for the entire population using FIT120. Incremental net monetary benefit is based on a cost-effectiveness threshold of £20,000/QALY.


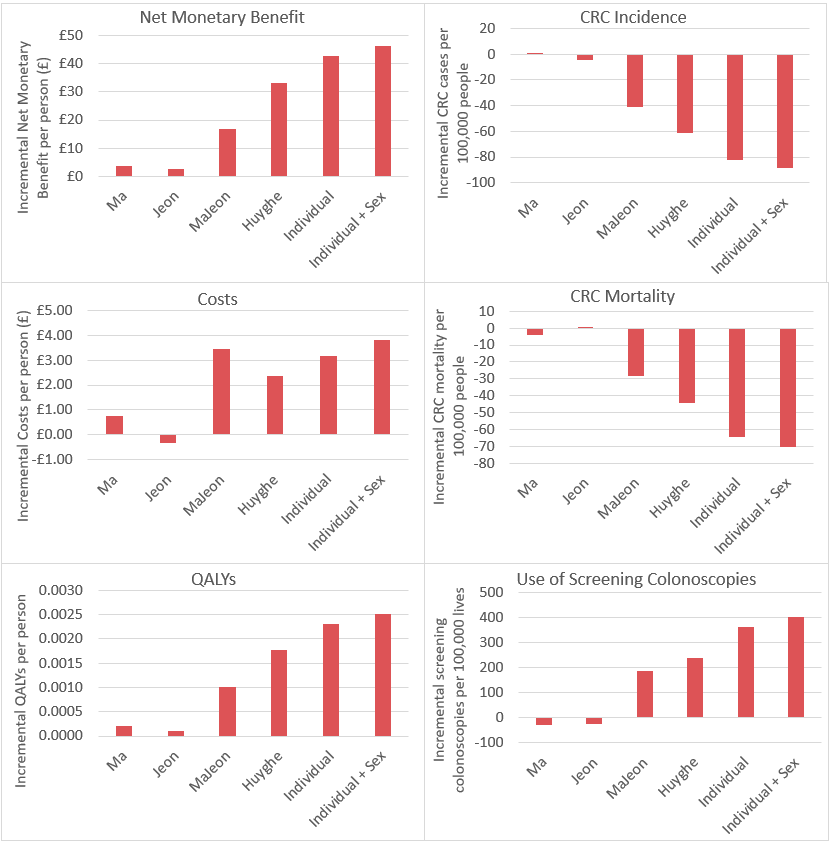


Supplementary Figure 3: Incremental outcomes for risk-stratification (based on a mean screening start age of 60), compared to screening start age of 60 for the entire population using FIT20. Incremental net monetary benefit is based on a cost-effectiveness threshold of £20,000/QALY.


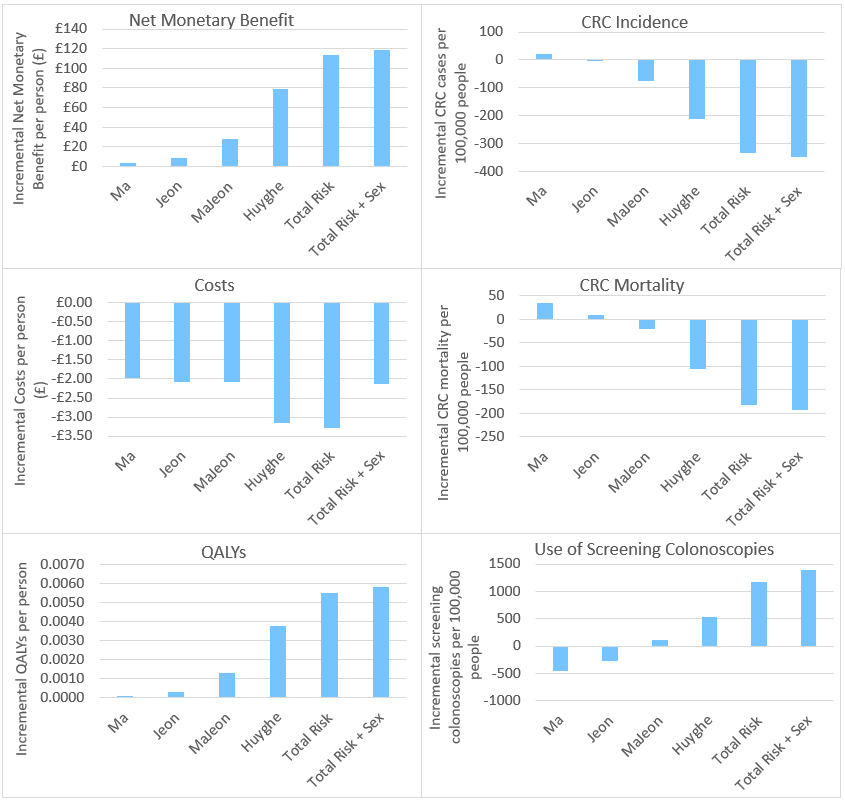


Supplementary Table 1: Values used in the model for FIT sensitivity and specificity (first round of screening).

| **FIT Threshold** | **Age** | **Sex** | **Sensitivity to CRC** | **Sensitivity to High Risk Adenoma** | **Sensitivity to Low Risk Adenoma** | **Specificity** |
| --- | --- | --- | --- | --- | --- | --- |
| FIT120 | 60 | Male | 0.354 | 0.104 | 0.020 | 0.996 |
|  |  | Female | 0.156 | 0.085 | 0.011 | 0.997 |
|  | 50 | Male | 0.356 | 0.080 | 0.014 | 0.998 |
|  |  | Female | 0.144 | 0.074 | 0.008 | 0.998 |
| FIT20 | 60 | Male | 0.410 | 0.249 | 0.080 | 0.976 |
|  |  | Female | 0.211 | 0.229 | 0.049 | 0.979 |
| FIT Faecal Immunochemical Test; CRC Colorectal Cancer | | | | | | |

Supplementary Table 2: Full set of incremental outcomes for risk-stratification based on a mean FIT120 screening start age of 50, compared to screening everyone with FIT120 at age 50.

| **Risk Score** | | **Ma** | **Jeon** | **MaJeon** | **Huyghe** | **Total Risk** | **Total Risk + Sex** |
| --- | --- | --- | --- | --- | --- | --- | --- |
| **Total Costs per person** | M | £0.78 | -£0.34 | £3.46 | £2.35 | £3.15 | £3.82 |
|  | *L* | *-£19* | *-£21* | *-£16* | *-£20* | *-£20* | *-£20* |
|  | *U* | *£20* | *£19* | *£26* | *£26* | *£27* | *£27* |
| **CRC Treatment Costs per person** | M | £0.54 | -£0.49 | -£1.23 | -£1.87 | -£2.51 | -£2.38 |
|  | *L* | *-£20* | *-£23* | *-£23* | *-£26* | *-£27* | *-£28* |
|  | *U* | *£20* | *£20* | *£22* | *£21* | *£23* | *£23* |
| **Screen & Surveillance Costs per person** | M | £0.23 | £0.15 | £4.69 | £4.22 | £5.67 | £6.20 |
|  | *L* | *-£5* | *-£5* | *-£1* | *-£1* | *£0* | *£0* |
|  | *U* | *£5* | *£5* | *£10* | *£10* | *£12* | *£13* |
| **QALYs per person** | M | 0.0002 | 0.0001 | 0.0010 | 0.0018 | 0.0023 | 0.0025 |
|  | *L* | *-0.0048* | *-0.0043* | *-0.0044* | *-0.0036* | *-0.0038* | *-0.0035* |
|  | *U* | *0.0050* | *0.0045* | *0.0059* | *0.0075* | *0.0087* | *0.0090* |
| **NMB per person (£20,000/QALY Threshold)** | M | £3.53 | £2.48 | £16.91 | £33.15 | £42.79 | £46.32 |
|  | *L* | *-£91* | *-£86* | *-£88* | *-£75* | *-£75* | *-£68* |
|  | *U* | *£96* | *£90* | *£112* | *£140* | *£171* | *£180* |
| **NMB per person (£30,000/QALY Threshold)** | M | £5.68 | £3.55 | £27.10 | £50.90 | £65.76 | £71.38 |
|  | *L* | *-£139* | *-£129* | *-£133* | *-£118* | *-£110* | *-£109* |
|  | *U* | *£149* | *£136* | *£172* | *£209* | *£257* | *£269* |
| **MJC per person (£20,000/QALY Threshold)** | M | £4.98 | £3.50 | £23.86 | £46.76 | £60.36 | £65.33 |
|  | *L* | *-£129* | *-£121* | *-£124* | *-£106* | *-£106* | *-£96* |
|  | *U* | *£136* | *£127* | *£158* | *£198* | *£241* | *£253* |
| **MJC per person (£30,000/QALY Threshold)** | M | £8.01 | £5.00 | £38.23 | £71.80 | £92.76 | £100.69 |
|  | *L* | *-£196* | *-£182* | *-£187* | *-£167* | *-£155* | *-£153* |
|  | *U* | *£210* | *£192* | *£243* | *£295* | *£363* | *£380* |
| **Prob. cost-effective (£20,000/QALY)** |  | 53% | 53% | 64% | 73% | 75% | 78% |
| **Prob. cost-effective (£30,000/QALY)** |  | 53% | 52% | 66% | 74% | 75% | 78% |
| **CRC Incidence per 100,000 people** | M | 1 | -5 | -41 | -61 | -83 | -89 |
|  | *L* | *-1106* | *-1098* | *-1201* | *-1242* | *-1324* | *-1363* |
|  | *U* | *1092* | *1050* | *1082* | *1121* | *1157* | *1165* |
| **CRC Mortality per 100,000 people** | M | -4 | 0 | -28 | -44 | -64 | -70 |
|  | *L* | *-483* | *-462* | *-518* | *-560* | *-616* | *-611* |
|  | *U* | *460* | *458* | *450* | *456* | *471* | *460* |
| **FIT screening invites per person** | M | -0.035 | -0.031 | 0.572 | 0.044 | 0.012 | 0.035 |
|  | *L* | *-0.058* | *-0.055* | *0.546* | *0.006* | *-0.091* | *-0.072* |
|  | *U* | *-0.012* | *-0.008* | *0.596* | *0.082* | *0.093* | *0.125* |
| **Screening colonoscopies per 100,000 people** | M | -28 | -26 | 188 | 237 | 360 | 403 |
|  | *L* | *-785* | *-782* | *-485* | *-517* | *-458* | *-362* |
|  | *U* | *723* | *728* | *916* | *1019* | *1206* | *1272* |
| QALY Quality Adjusted Life Year; NMB Net Monetary Benefit; MJC Maximum Justifiable Cost (of risk scoring procedure); CRC Colorectal Cancer; FIT Faecal Immunochemical Test; Prob. Probability; M Mean; L Lower 95% credible interval; U Upper 95% credible interval. | | | | | | | |

Supplementary Table 3: Full set of incremental outcomes for risk-stratification based on a mean FIT20 screening start age of 60, compared to screening everyone with FIT20 at age 60.

| **Risk Score** | | **Ma** | **Jeon** | **MaJeon** | **Huyghe** | **Total Risk** | **Total Risk + Sex** |
| --- | --- | --- | --- | --- | --- | --- | --- |
| **Total Costs per person** | M | -£1.96 | -£2.07 | -£2.08 | -£3.15 | -£3.29 | -£2.13 |
|  | *L* | *-£23* | *-£20* | *-£23* | *-£26* | *-£25* | *-£26* |
|  | *U* | *£17* | *£15* | *£16* | *£18* | *£20* | *£21* |
| **CRC Treatment Costs per person** | M | -£2.78 | -£2.67 | -£7.46 | -£14.36 | -£20.08 | -£20.48 |
|  | *L* | *-£24* | *-£22* | *-£30* | *-£40* | *-£44* | *-£47* |
|  | *U* | *£17* | *£16* | *£12* | *£8* | *£4* | *£5* |
| **Screen & Surveillance Costs per person** | M | £0.82 | £0.60 | £5.37 | £11.22 | £16.79 | £18.36 |
|  | *L* | *-£5* | *-£5* | *£0* | *£5* | *£9* | *£11* |
|  | *U* | *£6* | *£6* | *£12* | *£18* | *£25* | *£28* |
| **QALYs per person** | M | 0.0001 | 0.0003 | 0.0013 | 0.0038 | 0.0055 | 0.0058 |
|  | *L* | *-0.0043* | *-0.0037* | *-0.0029* | *-0.0007* | *0.0004* | *0.0007* |
|  | *U* | *0.0041* | *0.0041* | *0.0057* | *0.0091* | *0.0113* | *0.0118* |
| **NMB per person (£20,000/QALY Threshold)** | M | £3.77 | £8.36 | £28.10 | £79.03 | £113.32 | £118.45 |
|  | *L* | *-£78* | *-£73* | *-£57* | *-£14* | *£11* | *£20* |
|  | *U* | *£86* | *£86* | *£117* | *£181* | *£225* | *£233* |
| **NMB per person (£30,000/QALY Threshold)** | M | £4.68 | £11.50 | £41.11 | £116.97 | £168.33 | £176.60 |
|  | *L* | *-£120* | *-£109* | *-£87* | *-£17* | *£16* | *£30* |
|  | *U* | *£127* | *£126* | *£172* | *£271* | *£336* | *£357* |
| **MJC per person (£20,000/QALY Threshold)** | M | £5.32 | £11.79 | £39.64 | £111.48 | £159.85 | £167.08 |
|  | *L* | *-£110* | *-£103* | *-£81* | *-£20* | *£16* | *£28* |
|  | *U* | *£121* | *£121* | *£165* | *£256* | *£317* | *£329* |
| **MJC per person (£30,000/QALY Threshold)** | M | £6.59 | £16.22 | £58.00 | £165.00 | £237.45 | £249.12 |
|  | *L* | *-£169* | *-£154* | *-£123* | *-£24* | *£22* | *£43* |
|  | *U* | *£179* | *£177* | *£243* | *£382* | *£474* | *£503* |
| **Prob. cost-effective (£20,000/QALY)** |  | 54% | 57% | 74% | 95% | 99% | 99% |
| **Prob. cost-effective (£30,000/QALY)** |  | 53% | 57% | 74% | 94% | 99% | 98% |
| **CRC Incidence per 100,000 people** | M | 20 | -3 | -75 | -212 | -335 | -347 |
|  | *L* | *-1147* | *-1104* | *-1271* | *-1445* | *-1614* | *-1605* |
|  | *U* | *1210* | *1145* | *1090* | *984* | *923* | *886* |
| **CRC Mortality per 100,000 people** | M | 34 | 10 | -21 | -106 | -182 | -193 |
|  | *L* | *-480* | *-469* | *-536* | *-622* | *-709* | *-733* |
|  | *U* | *582* | *524* | *500* | *420* | *351* | *328* |
| **FIT screening invites per person** | M | 0.016 | -0.024 | 0.253 | 0.020 | 0.027 | 0.049 |
|  | *L* | *-0.011* | *-0.050* | *0.224* | *-0.022* | *-0.040* | *-0.018* |
|  | *U* | *0.043* | *0.002* | *0.283* | *0.061* | *0.088* | *0.112* |
| **Screening colonoscopies per 100,000 people** | M | -457 | -274 | 119 | 526 | 1166 | 1397 |
|  | *L* | *-1536* | *-1339* | *-1006* | *-762* | *-107* | *137* |
|  | *U* | *718* | *858* | *1302* | *1798* | *2448* | *2722* |
| QALY Quality Adjusted Life Year; NMB Net Monetary Benefit; MJC Maximum Justifiable Cost (of risk scoring procedure); CRC Colorectal Cancer; FIT Faecal Immunochemical Test; Prob. Probability; M Mean; L Lower 95% credible interval; U Upper 95% credible interval. | | | | | | | |

Supplementary Table 4: Full set of incremental outcomes for a scenario in which screening uptake is reduced by 25%. Risk-stratification based on a mean FIT120 screening start age of 60 is compared to screening everyone with FIT120 at age 60.

| **Risk Score** | | **Ma** | **Jeon** | **MaJeon** | **Huyghe** | **Total Risk** | **Total Risk + Sex** |
| --- | --- | --- | --- | --- | --- | --- | --- |
| **Total Costs per person** | M | -£1.97 | -£1.82 | -£1.24 | -£0.03 | £2.00 | £2.52 |
|  | *L* | *-£17* | *-£17* | *-£16* | *-£16* | *-£16* | *-£15* |
|  | *U* | *£13* | *£13* | *£14* | *£16* | *£20* | *£22* |
| **CRC Treatment Costs per person** | M | -£2.11 | -£1.58 | -£2.44 | -£4.91 | -£5.38 | -£5.69 |
|  | *L* | *-£18* | *-£17* | *-£18* | *-£22* | *-£24* | *-£25* |
|  | *U* | *£14* | *£14* | *£13* | *£12* | *£13* | *£14* |
| **Screen & Surveillance Costs per person** | M | £0.14 | -£0.24 | £1.19 | £4.88 | £7.38 | £8.21 |
|  | *L* | *-£3* | *-£4* | *-£2* | *£1* | *£3* | *£4* |
|  | *U* | *£4* | *£3* | *£5* | *£9* | *£12* | *£14* |
| **QALYs per person** | M | 0.0000 | 0.0001 | 0.0004 | 0.0019 | 0.0028 | 0.0031 |
|  | *L* | *-0.0033* | *-0.0031* | *-0.0030* | *-0.0017* | *-0.0011* | *-0.0008* |
|  | *U* | *0.0033* | *0.0033* | *0.0038* | *0.0060* | *0.0077* | *0.0078* |
| **NMB per person (£20,000/QALY Threshold)** | M | £1.33 | £3.80 | £9.74 | £37.75 | £54.60 | £59.20 |
|  | *L* | *-£61* | *-£55* | *-£56* | *-£31* | *-£21* | *-£14* |
|  | *U* | *£68* | *£68* | *£77* | *£119* | *£146* | *£150* |
| **NMB per person (£30,000/QALY Threshold)** | M | £1.01 | £4.78 | £13.98 | £56.61 | £82.90 | £90.06 |
|  | *L* | *-£91* | *-£86* | *-£82* | *-£50* | *-£31* | *-£20* |
|  | *U* | *£101* | *£102* | *£115* | *£179* | *£224* | *£232* |
| **MJC per person (£20,000/QALY Threshold)** | M | £1.87 | £5.36 | £13.73 | £53.25 | £77.02 | £83.51 |
|  | *L* | *-£86* | *-£77* | *-£79* | *-£43* | *-£29* | *-£19* |
|  | *U* | *£95* | *£96* | *£109* | *£168* | *£206* | *£211* |
| **MJC per person (£30,000/QALY Threshold)** | M | £1.42 | £6.75 | £19.72 | £79.85 | £116.94 | £127.04 |
|  | *L* | *-£128* | *-£122* | *-£116* | *-£70* | *-£43* | *-£28* |
|  | *U* | *£142* | *£144* | *£162* | *£252* | *£315* | *£327* |
| **Prob. cost-effective (£20,000/QALY)** |  | 50% | 54% | 61% | 86% | 93% | 93% |
| **Prob. cost-effective (£30,000/QALY)** |  | 50% | 54% | 61% | 87% | 93% | 93% |
| **CRC Incidence per 100,000 people** | M | 6 | 3 | -13 | -102 | -147 | -163 |
|  | *L* | *-978* | *-932* | *-1010* | *-1121* | *-1236* | *-1228* |
|  | *U* | *995* | *971* | *979* | *895* | *909* | *877* |
| **CRC Mortality per 100,000 people** | M | 30 | 21 | 12 | -56 | -102 | -115 |
|  | *L* | *-405* | *-394* | *-411* | *-498* | *-553* | *-579* |
|  | *U* | *458* | *426* | *438* | *384* | *329* | *344* |
| **FIT screening invites per person** | M | -0.176 | -0.221 | -0.160 | -0.139 | -0.115 | -0.081 |
|  | *L* | *-0.200* | *-0.239* | *-0.186* | *-0.173* | *-0.177* | *-0.146* |
|  | *U* | *-0.154* | *-0.200* | *-0.132* | *-0.103* | *-0.053* | *-0.020* |
| **Screening colonoscopies per 100,000 people** | M | -229 | -200 | -134 | 228 | 489 | 601 |
|  | *L* | *-807* | *-779* | *-725* | *-412* | *-187* | *-55* |
|  | *U* | *365* | *357* | *473* | *933* | *1161* | *1289* |
| QALY Quality Adjusted Life Year; NMB Net Monetary Benefit; MJC Maximum Justifiable Cost (of risk scoring procedure); CRC Colorectal Cancer; FIT Faecal Immunochemical Test; Prob. Probability; M Mean; L Lower 95% credible interval; U Upper 95% credible interval. | | | | | | | |

Supplementary Table 5: Incremental cost-effectiveness outcomes for risk-stratification based on a mean FIT120 screening start age of 60, compared to screening everyone with FIT120 at age 60, using a discount rate of 1.5%. Note that clinical and resource use outcomes are not discounted so are identical to those shown in Table 2 of the main results.

| **Risk Score** | | **Ma** | **Jeon** | **MaJeon** | **Huyghe** | **Total Risk** | **Total Risk + Sex** |
| --- | --- | --- | --- | --- | --- | --- | --- |
| **Total Costs per person** | M | -£1.42 | -£1.24 | -£0.68 | £1.24 | £3.00 | £3.64 |
|  | *L* | *-£11* | *-£11* | *-£10* | *-£9* | *-£9* | *-£8* |
|  | *U* | *£8* | *£8* | *£9* | *£12* | *£15* | *£16* |
| **CRC Treatment Costs per person** | M | -£1.79 | -£1.27 | -£2.01 | -£3.26 | -£3.73 | -£3.82 |
|  | *L* | *-£12* | *-£11* | *-£12* | *-£14* | *-£16* | *-£16* |
|  | *U* | *£8* | *£9* | *£8* | *£8* | *£9* | *£8* |
| **Screen & Surveillance Costs per person** | M | £0.37 | £0.03 | £1.33 | £4.51 | £6.72 | £7.46 |
|  | *L* | *-£2* | *-£2* | *-£1* | *£2* | *£3* | *£4* |
|  | *U* | *£3* | *£2* | *£4* | *£8* | *£10* | *£12* |
| **QALYs per person** | M | 0.0000 | 0.0001 | 0.0003 | 0.0013 | 0.0020 | 0.0022 |
|  | *L* | *-0.0018* | *-0.0016* | *-0.0015* | *-0.0007* | *-0.0002* | *-0.0003* |
|  | *U* | *0.0019* | *0.0019* | *0.0023* | *0.0037* | *0.0048* | *0.0048* |
| **NMB per person (£20,000/QALY Threshold)** | M | £1.01 | £2.70 | £7.00 | £25.31 | £36.62 | £39.69 |
|  | *L* | *-£33* | *-£30* | *-£29* | *-£16* | *-£9* | *-£5* |
|  | *U* | *£39* | *£37* | *£49* | *£69* | *£91* | *£91* |
| **NMB per person (£30,000/QALY Threshold)** | M | £0.81 | £3.42 | £10.16 | £38.59 | £56.43 | £61.36 |
|  | *L* | *-£49* | *-£45* | *-£42* | *-£23* | *-£10* | *-£6* |
|  | *U* | *£58* | *£53* | *£71* | *£106* | *£140* | *£139* |
| **MJC per person (£20,000/QALY Threshold)** | M | £1.65 | £4.39 | £11.41 | £41.23 | £59.66 | £64.66 |
|  | *L* | *-£54* | *-£49* | *-£47* | *-£27* | *-£15* | *-£9* |
|  | *U* | *£63* | *£61* | *£79* | *£112* | *£148* | *£148* |
| **MJC per person (£30,000/QALY Threshold)** | M | £1.32 | £5.58 | £16.56 | £62.85 | £91.93 | £99.95 |
|  | *L* | *-£80* | *-£74* | *-£68* | *-£38* | *-£16* | *-£10* |
|  | *U* | *£94* | *£87* | *£115* | *£173* | *£229* | *£226* |
| **Prob cost-effective (£20,000/QALY)** |  | 50% | 56% | 63% | 88% | 95% | 95% |
| **Prob cost-effective (£30,000/QALY)** |  | 49% | 56% | 62% | 88% | 95% | 95% |
| QALY Quality Adjusted Life Year; NMB Net Monetary Benefit; MJC Maximum Justifiable Cost (of risk scoring procedure); CRC Colorectal Cancer; FIT Faecal Immunochemical Test; Prob. Probability; M Mean; L Lower 95% credible interval; U Upper 95% credible interval. | | | | | | | |

Supplementary Table 6: Incremental cost-effectiveness outcomes for risk-stratification based on a mean FIT120 screening start age of 60, compared to screening everyone with FIT120 at age 60, using a discount rate of 5%. Note that clinical and resource use outcomes are not discounted so are identical to those shown in Table 2 of the main results.

| **Risk Score** | | **Ma** | **Jeon** | **MaJeon** | **Huyghe** | **Total Risk** | **Total Risk + Sex** |
| --- | --- | --- | --- | --- | --- | --- | --- |
| **Total Costs per person** | M | -£8.19 | -£6.54 | -£8.18 | -£10.16 | -£10.08 | -£9.52 |
|  | *L* | *-£45* | *-£45* | *-£47* | *-£48* | *-£54* | *-£51* |
|  | *U* | *£29* | *£33* | *£31* | *£29* | *£34* | *£33* |
| **CRC Treatment Costs per person** | M | -£6.68 | -£4.86 | -£9.05 | -£18.98 | -£24.27 | -£25.77 |
|  | *L* | *-£46* | *-£46* | *-£51* | *-£59* | *-£70* | *-£68* |
|  | *U* | *£33* | *£35* | *£33* | *£24* | *£22* | *£19* |
| **Screen & Surveillance Costs per person** | M | -£1.51 | -£1.68 | £0.87 | £8.82 | £14.19 | £16.25 |
|  | *L* | *-£11* | *-£11* | *-£9* | *-£1* | *£3* | *£5* |
|  | *U* | *£8* | *£7* | *£10* | *£20* | *£27* | *£29* |
| **QALYs per person** | M | -0.0007 | 0.0000 | 0.0009 | 0.0058 | 0.0087 | 0.0096 |
|  | *L* | *-0.0098* | *-0.0094* | *-0.0091* | *-0.0048* | *-0.0026* | *-0.0022* |
|  | *U* | *0.0097* | *0.0097* | *0.0114* | *0.0169* | *0.0212* | *0.0222* |
| **NMB per person (£20,000/QALY Threshold)** | M | -£6.23 | £6.07 | £26.30 | £125.93 | £184.21 | £202.11 |
|  | *L* | *-£190* | *-£179* | *-£166* | *-£84* | *-£47* | *-£22* |
|  | *U* | *£196* | *£199* | *£233* | *£345* | *£424* | *£452* |
| **NMB per person (£30,000/QALY Threshold)** | M | -£13.44 | £5.83 | £35.36 | £183.81 | £271.27 | £298.41 |
|  | *L* | *-£290* | *-£272* | *-£255* | *-£132* | *-£68* | *-£52* |
|  | *U* | *£296* | *£301* | *£344* | *£517* | *£639* | *£675* |
| **MJC per person (£20,000/QALY Threshold)** | M | -£7.23 | £7.04 | £30.52 | £146.14 | £213.78 | £234.56 |
|  | *L* | *-£220* | *-£208* | *-£193* | *-£97* | *-£55* | *-£26* |
|  | *U* | *£227* | *£231* | *£271* | *£400* | *£492* | *£525* |
| **MJC per person (£30,000/QALY Threshold)** | M | -£15.59 | £6.76 | £41.04 | £213.32 | £314.82 | £346.31 |
|  | *L* | *-£336* | *-£316* | *-£296* | *-£153* | *-£79* | *-£60* |
|  | *U* | *£344* | *£350* | *£399* | *£600* | *£742* | *£783* |
| **Prob cost-effective (£20,000/QALY)** |  | 46% | 53% | 57% | 87% | 95% | 95% |
| **Prob cost-effective (£30,000/QALY)** |  | 45% | 52% | 56% | 86% | 95% | 95% |
| QALY Quality Adjusted Life Year; NMB Net Monetary Benefit; MJC Maximum Justifiable Cost (of risk scoring procedure); CRC Colorectal Cancer; FIT Faecal Immunochemical Test; Prob. Probability; M Mean; L Lower 95% credible interval; U Upper 95% credible interval. | | | | | | | |

Supplementary Table 7: Full set of incremental outcomes by sex for risk-stratification based on a mean FIT120 screening start age of 60, compared to screening everyone with FIT120 at age 60.

| **Risk Score** | | **Ma** | **Jeon** | **MaJeon** | **Huyghe** | **Total Risk** | **Total Risk + Sex** |
| --- | --- | --- | --- | --- | --- | --- | --- |
| **Males** | | | | | | | |
| **Total Costs per person** | M | -£3.40 | -£3.23 | -£2.76 | -£0.42 | £1.59 | £6.31 |
|  | *L* | *-£33* | *-£31* | *-£32* | *-£31* | *-£33* | *-£30* |
|  | *U* | *£25* | *£23* | *£28* | *£31* | *£39* | *£40* |
| **CRC Treatment Costs per person** | M | -£4.47 | -£2.84 | -£5.57 | -£7.94 | -£10.87 | -£11.96 |
|  | *L* | *-£34* | *-£32* | *-£35* | *-£42* | *-£49* | *-£52* |
|  | *U* | *£24* | *£25* | *£26* | *£27* | *£26* | *£26* |
| **Screen & Surveillance Costs per person** | M | £1.07 | -£0.39 | £2.81 | £7.53 | £12.46 | £18.27 |
|  | *L* | *-£6* | *-£7* | *-£4* | *£0* | *£3* | *£8* |
|  | *U* | *£8* | *£7* | *£10* | *£17* | *£23* | *£30* |
| **QALYs per person** | M | 0.0002 | 0.0000 | 0.0009 | 0.0029 | 0.0047 | 0.0061 |
|  | *L* | *-0.0060* | *-0.0055* | *-0.0048* | *-0.0037* | *-0.0026* | *-0.0016* |
|  | *U* | *0.0062* | *0.0062* | *0.0077* | *0.0101* | *0.0134* | *0.0152* |
| **NMB per person (£20,000/QALY Threshold)** | M | £7.32 | £4.03 | £20.01 | £59.22 | £91.56 | £114.95 |
|  | *L* | *-£115* | *-£107* | *-£92* | *-£66* | *-£53* | *-£31* |
|  | *U* | *£132* | *£118* | *£149* | *£205* | *£262* | *£302* |
| **NMB per person (£30,000/QALY Threshold)** | M | £9.27 | £4.42 | £28.64 | £88.62 | £138.14 | £175.58 |
|  | *L* | *-£174* | *-£158* | *-£141* | *-£99* | *-£82* | *-£45* |
|  | *U* | *£197* | *£179* | *£218* | *£304* | *£391* | *£456* |
| **MJC per person (£20,000/QALY Threshold)** | M | M | £5.68 | £28.23 | £83.53 | £129.16 | £162.15 |
|  | *L* | *-£162* | *-£151* | *-£130* | *-£94* | *-£74* | *-£43* |
|  | *U* | *£186* | *£166* | *£210* | *£289* | *£370* | *£427* |
| **MJC per person (£30,000/QALY Threshold)** | M | £13.08 | £6.24 | £40.39 | £125.01 | £194.86 | £247.67 |
|  | *L* | *-£245* | *-£223* | *-£198* | *-£140* | *-£115* | *-£63* |
|  | *U* | *£278* | *£253* | *£307* | *£429* | *£552* | *£643* |
| **CRC Incidence per 100,000 people** | M | -3 | 19 | -29 | -132 | -229 | -321 |
|  | *L* | *-1878* | *-1758* | *-1879* | *-2021* | *-2175* | *-2310* |
|  | *U* | *1867* | *1837* | *1878* | *1728* | *1734* | *1625* |
| **CRC Mortality per 100,000 people** | M | 44 | 41 | 20 | -65 | -134 | -219 |
|  | *L* | *-707* | *-685* | *-754* | *-808* | *-945* | *-1039* |
|  | *U* | *849* | *788* | *822* | *697* | *639* | *555* |
| **FIT screening invites per person** | M | -0.008 | -0.203 | 0.019 | -0.121 | 0.008 | 0.930 |
|  | *L* | *-0.049* | *-0.263* | *-0.040* | *-0.226* | *-0.150* | *0.771* |
|  | *U* | *0.032* | *-0.151* | *0.076* | *-0.002* | *0.166* | *1.085* |
| **Screening colonoscopies per 100,000 people** | M | -334 | -345 | -170 | 317 | 820 | 1455 |
|  | *L* | *-1543* | *-1577* | *-1308* | *-873* | *-413* | *50* |
|  | *U* | *920* | *856* | *1141* | *1565* | *2226* | *2856* |
| **Females** | | | | | | | |
| **Total Costs per person** | M | £0.45 | £0.41 | £0.17 | -£2.85 | £1.36 | -£1.11 |
|  | *L* | *-£21* | *-£18* | *-£20* | *£6* | *-£20* | *-£23* |
|  | *U* | *£20* | *£19* | *£19* | *£7* | *£23* | *£21* |
| **CRC Treatment Costs per person** | M | £0.09 | -£0.68 | -£1.31 | -£4.91 | -£6.37 | -£5.29 |
|  | *L* | *-£22* | *-£21* | *-£22* | *-£28* | *-£29* | *-£28* |
|  | *U* | *£21* | *£18* | *£18* | *£18* | *£17* | *£18* |
| **Screen & Surveillance Costs per person** | M | £0.36 | £1.09 | £1.48 | £6.15 | £7.73 | £4.18 |
|  | *L* | *-£4* | *-£3* | *-£3* | *£1* | *£2* | *-£1* |
|  | *U* | *£5* | *£5* | *£6* | *£12* | *£14* | *£10* |
| **QALYs per person** | M | 0.0000 | 0.0003 | 0.0005 | 0.0024 | 0.0031 | 0.0024 |
|  | *L* | *-0.0042* | *-0.0042* | *-0.0045* | *-0.0027* | *-0.0019* | *-0.0029* |
|  | *U* | *0.0042* | *0.0049* | *0.0058* | *0.0082* | *0.0093* | *0.0084* |
| **NMB per person (£20,000/QALY Threshold)** | M | £0.34 | £5.90 | £9.94 | £45.79 | £60.22 | £49.59 |
|  | *L* | *-£84* | *-£77* | *-£87* | *-£46* | *-£36* | *-£48* |
|  | *U* | *£81* | *£92* | *£109* | *£158* | *£182* | *£169* |
| **NMB per person (£30,000/QALY Threshold)** | M | £0.74 | £9.05 | £15.00 | £69.30 | £91.01 | £73.83 |
|  | *L* | *-£125* | *-£119* | *-£130* | *-£71* | *-£51* | *-£73* |
|  | *U* | *£124* | *£141* | *£163* | *£237* | *£279* | *£250* |
| **MJC per person (£20,000/QALY Threshold)** | M | £0.48 | £8.32 | £14.03 | £64.59 | £84.95 | £69.95 |
|  | *L* | *-£119* | *-£109* | *-£123* | *-£65* | *-£50* | *-£68* |
|  | *U* | *£115* | *£129* | *£153* | *£223* | *£257* | *£239* |
| **MJC per person (£30,000/QALY Threshold)** | M | £1.04 | £12.76 | £21.16 | £97.75 | £128.38 | £104.14 |
|  | *L* | *-£177* | *-£167* | *-£184* | *-£101* | *-£72* | *-£103* |
|  | *U* | *£174* | *£199* | *£230* | *£334* | *£394* | *£353* |
| **CRC Incidence per 100,000 people** | M | 7 | -21 | -28 | -137 | -183 | -123 |
|  | *L* | *-1228* | *-1281* | *-1267* | *-1436* | *-1522* | *-1497* |
|  | *U* | *1205* | *1220* | *1252* | *1202* | *1196* | *1254* |
| **CRC Mortality per 100,000 people** | M | -7 | -21 | -25 | -108 | -145 | -98 |
|  | *L* | *-559* | *-575* | *-600* | *-712* | *-774* | *-731* |
|  | *U* | *546* | *535* | *569* | *492* | *468* | *542* |
| **FIT screening invites per person** | M | 0.039 | 0.146 | 0.049 | 0.186 | 0.094 | -0.721 |
|  | *L* | *0.014* | *0.095* | *-0.002* | *0.082* | *-0.080* | *-0.891* |
|  | *U* | *0.064* | *0.192* | *0.101* | *0.282* | *0.262* | *-0.551* |
| **Screening colonoscopies per 100,000 people** | M | -8 | 126 | 87 | 493 | 642 | 303 |
|  | *L* | *-795* | *-648* | *-776* | *-310* | *-194* | *-476* |
|  | *U* | *778* | *895* | *893* | *1383* | *1490* | *1148* |
| QALY Quality Adjusted Life Year; NMB Net Monetary Benefit; MJC Maximum Justifiable Cost (of risk scoring procedure); CRC Colorectal Cancer; FIT Faecal Immunochemical Test; Prob. Probability; M Mean; L Lower 95% credible interval; U Upper 95% credible interval. | | | | | | | |
